# Molecular basis of contact inhibition of locomotion

**DOI:** 10.1007/s00018-015-2090-0

**Published:** 2015-11-19

**Authors:** Alice Roycroft, Roberto Mayor

**Affiliations:** Department of Cell and Developmental Biology, University College London, Gower Street, London, WC1E 6BT UK

**Keywords:** Cell migration, Cell polarity, Cell adhesion, Cadherin, Rho, Rac

## Abstract

Contact inhibition of locomotion (CIL) is a complex process, whereby cells undergoing a collision with another cell cease their migration towards the colliding cell. CIL has been identified in numerous cells during development including embryonic fibroblasts, neural crest cells and haemocytes and is the driving force behind a range of phenomenon including collective cell migration and dispersion. The loss of normal CIL behaviour towards healthy tissue has long been implicated in the invasion of cancer cells. CIL is a multi-step process that is driven by the tight coordination of molecular machinery. In this review, we shall breakdown CIL into distinct steps and highlight the key molecular mechanisms and components that are involved in driving each step of this process.

## Introduction

Contact inhibition of locomotion (CIL) is a multi-faceted process, whereby colliding cells that come into contact with each other cease their migration towards their colliding partner before repolarising and migrating away from each other. Leo Loeb initially observed this phenomenon in the 1920s among amoebocyte haemocytes in horseshoe crabs (*Limulus*) where he noted that the haemocytes ‘move toward each other and meet and stick together. Subsequently, the agglutinating cells send out pseudopods in such a way that the cells become again separated from each other’ [1]. It was not until the 1950s, however, that CIL was properly characterised by the influential cell and developmental biologist, Michael Abercrombie [2]. Abercrombie was interested in the social behaviour of cells, i.e. how a cell is influenced by the other cells in its surrounding. His early observations of migrating chick heart embryonic fibroblasts revealed an interesting behaviour where the mean velocity of a single migrating cell was inversely proportional to the amount of contacts it made with other fibroblasts [3]. Abercrombie made more extensive observations of this behaviour and noted that not only was velocity restricted upon a collision with another cell, but the directionality was affected as well [3]. He coined the words ‘contact inhibition’ in order to describe this phenomenon where colliding cells cease migrating in the direction of the contact. CIL can occur when cells of the same type collide (homotypic CIL), or when cells of a different type collide (heterotypic CIL). In the 60 years following its initial characterisation in chick heart embryonic fibroblasts, homotypic CIL has been identified in a variety of other cells types including somitic cells [4], neural crest cells [4, 5], haemocytes [6], and Cajal–Retzius neurons [7]. Heterotypic CIL can occur between two cell types that independently show homotypic CIL, such as between fibroblasts and prostate cancer cells [8] and neural crest and somitic cells [9]. Heterotypic CIL can also occur between a cell type that shows homotypic CIL and one that does not such as between neural crest cells and placodes [10]. CIL is important in immobilising cells within a healthy tissue [11] and the loss of heterotypic CIL towards healthy tissue is implicated in metastasis and invasion in cancer [11–16]. In the developing embryo CIL is vital for the directional collective migration of the neural crest [5], the precise dispersion patterning of haemocytes [17] and the regular dispersion of Cajal–Retzius neurons throughout the cortex [7]. In addition, CIL appears to play a role in the contact-dependent polarity that drives the tightly coordinated migration of border cells in the *Drosophila* ovary [18, 19]. For many decades following its initial characterisation by Abercrombie, the molecular mechanisms underlying CIL remained unknown. Its discovery in the embryo [5] has led to a resurgence in the field of CIL and the molecular components that drive CIL have finally begun to be elucidated. This review shall discuss some of the molecular machinery that helps drive CIL. In order to do this we shall break CIL down into four discrete steps and highlight some of the key molecular mechanisms and components that are involved in each step of this process.

## Defining contact inhibition of locomotion

In the decade following Abercrombie’s initial discovery of CIL in fibroblasts, a density-dependent inhibition of cell growth was identified [20, 21]. This is a process whereby cells reduce their rate of proliferation when they become confluent; it is often referred to as contact inhibition. It is important to note that this contact inhibition of cell growth and replication is distinct from CIL and the mechanisms driving them are independent of each other [22]. The phenomenon of contact inhibition of cell growth will not be discussed further in this review, which focuses solely on contact inhibition of locomotion.

**Fig. 1:**
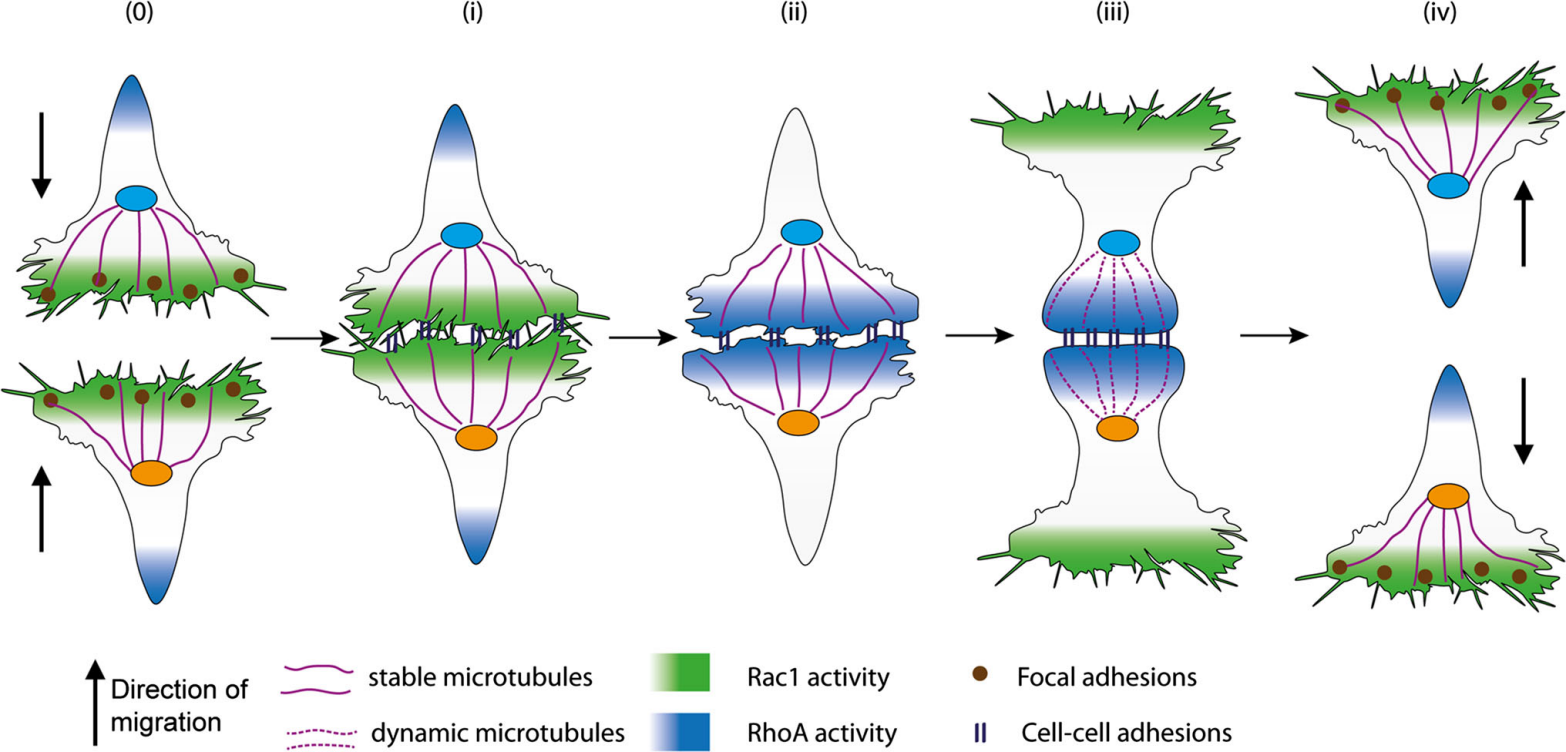
The multiply stages of contact inhibition of locomotion. a Free migrating cells show polarised migration: Rac1 activity in the leading edge stimulates protrusion formation. Microtubules stabilise the directional migration of these cells. In addition, focal adhesions generation traction forces enabling the cells to migrate along a substrate. b Initially a contact is formed between the cells: the lamellae of the colliding cells overlap and cell–cell adhesions form between the two cells. The cytoskeletons of the colliding cells become coupled. c Protrusive activity is inhibited at the site of contact: Rac1 activity is lost at the contact site and RhoA become active at the point. This causes the protrusions to collapse and prevents new protrusions from forming at the contact site. d The cells repolarise and new protrusions form away from the contact: Rac1 becomes active in the free edge away from the contact promoting the formation of new protrusions in this area. Focal adhesions form in these new protrusions and stabilises them. Microtubule dynamics increase at the contact site with an increase in growth and shrinkage rates and microtubule catastrophe events. e The cells separate and migrate away from each other: the cells continue migrating in the direction of the newly formed protrusions away from the direction of contact. The cell–cell adhesions disassemble and the cells final separate

The precise definition of CIL has evolved over time with the ever increasing understanding of this phenomenon. Initially Abercrombie defined CIL as ‘the prohibition, when contact between cells occurred, of continued movement such as would carry one cell over the surface of another’ [23]. This description is still the defining characteristic of CIL; however, more detailed observations of CIL in a variety of cell types have allowed this definition to be expanded. CIL is often subdivided into two categories: types I and II [24]. Type I, as first observed in fibroblasts by Abercrombie, is characterised by paralysis of membrane ruffling and a contraction at the leading edge [25]. Type II, as described by Carter, does not involve contraction of the leading edge; the cessation of migration in the direction of contact is inhibited solely due to the difficulty of the cell to migrate across the surface of the other cell [26]. Abercrombie himself questioned whether collisions without contraction at the leading edge, as observed in type II collisions, were in fact CIL, stating that type II collisions bear ‘little resemblance to contact inhibition’ [27] and many believe that contraction of the leading edge is a necessity for CIL [28]. The identification of the molecular mechanisms involved in type I CIL indicate that it is an active process and distinct from the more passive type II CIL. This review, therefore, will focus on type I CIL. A key characteristic of type I CIL is that an unrestricted cell upon a collision ceases ‘to continue moving in the same direction after contact with another cell’ [12]. Instead the cell repolarises and migrates away from the contact. A restricted cell, i.e. one that is completely surrounded by cells, such as those in a cluster, would have their protrusions inhibited on all sides [29, 30]. The process of CIL can be broken down into four discrete stages (Fig. 1): (1) initially a contact is formed between the cells; (2) protrusive activity is inhibited at the site of contact; (3) the cells repolarise and new protrusions form away from the contact; (4) the cells separate and migrate away from each other.

## Methods to study contact inhibition of locomotion

Upon its initial characterisation Abercrombie speculated about the importance of CIL in maintaining healthy tissue [11] and proposed how its loss towards healthy tissue could be a prerequisite for metastasis [11–13, 31]. In order to characterise CIL and better understand its role in cancer and development, several different assays have been developed over the years. Abercrombie first characterised CIL using a technique, whereby two chick heart explant cultures were plated between 0.5 and 1 mm apart [2]. The cells would grow out from these explants and their behaviour towards each other could be observed in the gap between them. He used this assay to characterise CIL and demonstrate that sarcoma cells lose CIL towards healthy fibroblasts [2, 3, 11, 25]. Similar techniques are still used to address whether cells are invasive towards chick heart explants [32] and 3D image reconstructions can give a more detailed view of the invasion taking place. A comparable confrontation assay was used to establish the role of CIL in the neural crest and the behaviour of explants towards each other could be observed [5, 10, 30, 33]. CIL between single cells has predominantly been characterised on 2D substrates [31, 34]. Individual cells migrate randomly and stochastic collisions between them are observed. This method has been used to investigate why cancerous cells lose CIL towards normal fibroblasts and has helped elucidate mechanisms controlling CIL [5, 8, 10, 16, 35–37]. Cells on a 2D substrate can collide from any incoming angle. It has long been established that head-to-head collisions show distinct CIL behaviour whereas other collisions, such as head-to-side where lamellae do no overlap, do not [17, 38]. In order to restrict cell–cell interactions to more reproducible head-to-head collisions, a 1D collision assay was generated [39, 40]. This method confines cells to micropatterned extracellular matrix lanes restricting the angle of collision to head-on only and forcing the cells to repolarise 180°. Forcing the cells to completely reverse their front–rear polarity makes it easier to establish the steps required for this repolarisation and the temporal regulation of these events. In addition, restricting the cells to 1D lanes makes it easier to predict when cells are going to interact and allows for easier analysis [39, 40]. An additional assay has been generated that restricts cells to 1D migration through the use of microchannels. In this assay microfluidic chambers constrain cell migration to 1D channels whilst allowing chemoattractant gradients to be generated across the chamber [41]. These chambers have proved useful in understanding how CIL is affected by chemotactic cues found in vivo [15]. This is of particular interest as cancer cells are known to migrate through tracks generated in the extracellular matrix [40] and respond to chemotactic cues [42, 43].

## Contact inhibition of locomotion in vivo

Contact inhibition of locomotion has been identified as the driving force behind many phenomena in developing embryos [44]. As with all in vitro assays, there is some uncertainty as to whether cells’ behaviour in vitro mimics their behaviour in vivo. This question has begun to be addressed thanks to the improvement in imaging of CIL in the developing embryo. Haemocytes undergoing CIL can be imaged in vivo in the ventral surface of *Drosophila* [6]. The behaviour observed between these cells is strikingly similar to what Abercrombie first observed in fibroblasts in vitro over 50 years earlier [2, 17]. Further evidence that CIL is similar in vivo has been observed in the zebrafish cranial neural crest where the trajectories of cells undergoing collisions in vivo are similar to those of cells in vitro [5]. These observations confirm that the in vitro assays are mimicking what is happening in vivo and are therefore useful in elucidating the molecular mechanisms driving CIL. The development and improvement of new live imaging techniques have helped elucidate some of the mechanisms driving CIL. In the haemocytes of *Drosophila* CIL occurs between individual cells and is required to drive the uniform dispersion of the haemocytes throughout the drosophila embryo [17]. Interestingly CIL drives a completely distinct process within the neural crest, where it is vital for their directional collective migration [5, 10, 30, 45]. It has been proposed that CIL contribute to collective migration of the neural crest by inhibiting protrusions forming within the cluster and driving the polarisation of the cells at the leading edge [29]. Interestingly it has been observed that CIL between single cells in a 1D environment can lead to persistent polarised chains of cells coherently migrating in a given direction [40]. The more cells in the chain the more persistent the migration of the collective of cells. Furthermore, it was also observed that cells within these chains became coupled together through cell–cell adhesions [40], suggesting CIL could be functionally linked to collective migration through the coupling of cell–cell adhesions. CIL is just one of the many factors that has to be carefully mediated for the collective migration of the neural crest [46], chemotaxis also plays a role in their collective migration [10, 30, 47]. It has recently been shown that the outcome of CIL collisions changes in the presence of a chemoattractant gradient [15]. When cells collide in the presence of a chemoattractant gradient they are more likely to repolarise in the direction of the chemoattractant, even if this means they are not polarising away from the contact. However, the outcome of a collision is dependent on the balance of CIL versus chemotactic response and can be shifted between one outcome or another depending on the signalling pathways activated [15]. In neural crest explants it appears that while CIL polarises the cells at the edge of the cluster away from the contact, the chemoattractant SDF1 stabilises these protrusions at the leading edge [10, 30]. Overall these experiments demonstrate how directional migration and CIL could work together to polarise the cells and drive collective migration. Although the role of CIL in collective migration has predominantly been studied in the neural crest, it is likely to play a similar role in the collective migration of other cell types.

## Molecular machinery driving contact inhibition of locomotion

Contact inhibition of locomotion is a complex process that involves many different molecular mechanisms. Each of the four distinct steps of CIL requires changes to the cytoskeleton driven by a variety of molecular components [36, 48, 49]. The following part of this review will break down the process of CIL into these four stages and highlight the key components involved in driving each step.

### A contact is formed between the cells

#### Formation of a cell–cell adhesion complex

It has long been established that the formation of a physical contact between colliding partners is a requirement for CIL and no changes occur in the lamellae prior to this event [25]. The fact that an adhesive contact must be forming between colliding cell partners was further evident by the observation that tension is generated in the lamellae across a contact [25, 28, 50]. After Abercrombie’s discovery of CIL in fibroblasts, work was done to elucidate the nature of these adhesions using the microscopy techniques available at the time. Heaysman and Pegrum coupled the behaviour of the adhesions to the different stages of CIL in fibroblasts [28]. They noted that cell–cell adhesions formed between colliding cells soon after a collision and speculated that the abrupt separation of the cells was due to the loss of these adhesions. Interestingly cell–cell adhesions were not observed when fibroblasts collided with sarcoma cells [51], where normal CIL behaviour is known to be lost [11]. Although the exact nature of these adhesions was speculated upon [23], the limitations of the microscopy and molecular biology techniques available prevented the identification of the molecular components involved. It was not until decades later that the nature of these adhesions could begin to be elucidated. One potentially surprising aspect of the cell–cell adhesions identified in CIL is that they do not all belong to the same family of adhesion complexes. This suggests that CIL may be driven through a variety of different mechanisms. We will discuss some of the adhesion molecules involved in CIL.

*Cadherins* The first family of cell–cell adhesion molecules to be identified in CIL were the cadherins [52]. Cadherins are a family of transmembrane glycoproteins that facilitate calcium-dependent cell–cell adhesions. They form adherens junctions between neighbouring cells and tightly regulate the actin cytoskeleton [53]. Their importance in CIL was first identified in L-cell lines where it was demonstrated that the presence of E-cadherin, the cadherin predominantly expressed in epithelial cells, caused paralysis of the lamellae upon a collision [52]. Furthermore, E-cadherin has since been identified as the adhesion molecule required to inhibit the protrusive activity and migration of confluent epithelial cells [54] and its disruption has been associated with the loss of this behaviour in carcinoma cells [55]. N-cadherin, the cadherin first discovered in the neural plate, is required for CIL in a variety of cell types [14, 30, 56]. In myoblasts and glial cells it is required for the cessation of migration and paralysis of lamellae upon a collision [14, 56]. In addition N-cadherin and cadherin-11 are essential for CIL between neural crest cells where their loss inhibits the migration of the neural crest in vivo [30, 57]. In vitro cultures of neural crest cells show normal CIL behaviour, where colliding cells form a contact, collapse protrusions and cease migration before repolarising and migrating away from each other. When either N-cadherin or cadherin-11 is inhibited the colliding neural crest no longer show normal CIL behaviour, instead they continue migrating in the direction of contact and no longer repolarise away from the contact. In addition, there is an increase in protrusive activity at the contact, indicating that the normal paralysis of lamellae is lost. Interestingly, blocking N-cadherin junctions in Schwann cells seems to promote a CIL like process, where the cells pull away from each other after coming into contact [58].

*Eph*-*ephrin* Another group of proteins that are known to mediate cell–cell interactions during CIL are the Eph receptors. These are a group of tyrosine kinase receptors that bind transmembrane ephrin ligands from the neighbouring cell and couple the cells upon cell–cell contact. The binding of the ligand by the receptor triggers bidirectional signalling cascades in both the ligand-expressing and the receptor-expressing cells [59]. Eph/ephrins are expressed in all germ layers. They are essential for many aspects of development including vascular and skeleton morphogenesis, boundary formation and axon guidance (as reviewed in [60]) and their dysregulation is associated with disease [61]. Interestingly Eph-ephrin mediated cell–cell interactions are often, but not always, associated with a repulsive response in the coupled cells causing the cells to retract upon contact in a process similar to CIL [62–64]. EphA signalling can facilitate CIL in prostate cancer cells by promoting a repulsive behaviour between cells [8, 35]; whereas, EphB signalling suppresses CIL and increases membrane ruffling at the site of contact by promoting cell–cell attraction [16, 64]. Interestingly, this difference in behaviour controlled by a shift in the balance of activities of EphA to EphB, is strikingly similar to the cadherin switch from E- to N- that dictates whether neural crest cells undergo CIL or not [33]. Both EphA and EphB are required for CIL in Cajal–Retzius neurons and to drive their proper dispersion [7]. EphB signalling gives rise to CIL in a carcinoma cell line and can induce high levels of CIL behaviour, which can override chemotactic cues [15]. Whether the full spectrum of cell–cell adhesion complexes that contribute to CIL have been identified is unknown. During CIL of haemocytes in *Drosophila* [6, 17, 48] zyxin has been shown to localise at the cell–cell contact [48]; however, the molecular nature of the cell adhesion molecule at the contact remains unknown. The engagement of this unidentified cell–cell adhesion is essential for CIL through its ability to couple the cytoskeletons in the colliding partners, allowing tension to be built up in their lamellae prior to separation [48].

### Protrusive activity is inhibited at the site of contact

#### Regulation of small GTPase activity

The distinct steps of CIL are each driven by cytoskeleton rearrangements and dynamics that in turn are controlled by the activity of Rho family GTPases [65]. RhoA and Rac1 are the best understood members of the RhoGTPases. The canonical understanding is that RhoA generates contraction through the regulation of actomyosin and activation of ROCK [66], while Rac1 drives the formation of lamellipodia [67] through the mediation of actin polymerisation. Here we highlight the RhoGTPases identified at the contact during CIL.

**Fig. 2:**
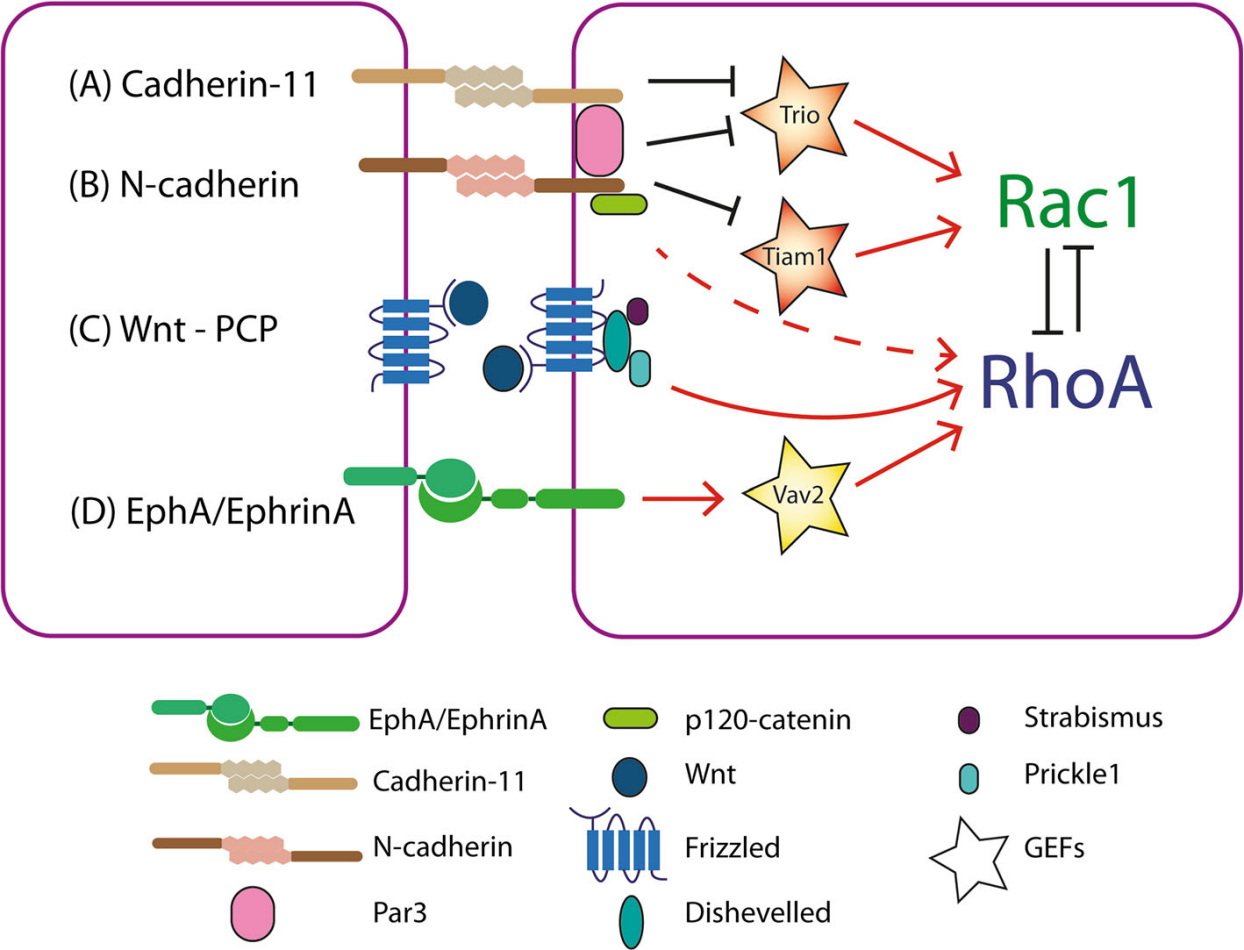
The RhoGTPase switch at the cell–cell contact. a Cadherin-11 sequesters Trio to the contact where it is inhibited. Trio activates Rac1 and inhibits RhoA. As Trio is sequestered and inhibited at the contact, Rac1 cannot be activated and the inhibition on RhoA is lifted. It is possible that Cadherin-11 inhibits Trio via the recruitment of the polarity protein Par3. b N-cadherin may be influencing the behaviour of the RhoGTPases through several means. One possibility is that it recruits Par3 to the contact and that in turn inhibits Trio. Secondly N-cadherin leads to the inhibition of the GEF—Tiam1 via its association with nm23. Nm23 binds and inhibits Tiam1 at the contact site. Tiam1 is an activator of Rac1 and its inhibition prevent the activation of Rac1 at the contact site. Interaction with p120-catenin is the determining factor influencing the differential behaviour of the RhoGTPases downstream of E- and N-cadherin. It is likely that p120-catenin is signalling through an as yet unidentified means leading to the activation of RhoA and inhibition of Rac1 at the contact. c The non-canonical Wnt-planar cell polarity pathway is activated by Wnt11 binding to the receptor Frizzled. Dishevelled, Prickle1 and Strabismus are recruited to the receptor at the contact upon a collision. The activation of this pathway results in the activation of RhoA near the contact. Due to the shared component p120-catenin it is possible N-cadherin binding stimulates signalling through the planar cell polarity pathway. d EphA binds EphrinA from the neighbouring cell. This stimulates bidirectional signalling that results in the activation of the GEF—Vav2. Vav2 in turn activates RhoA

One distinct feature of CIL is the paralysis of membrane ruffling and inhibition of protrusive activity at the leading edge upon a collision [25, 28, 30, 48, 68]. In a free migrating cell Rac1 is active in the leading edge. This drives actin polymerisation and subsequently protrusion formation at this site [67]. Upon a collision a switch in the activity of the RhoGTPases occurs at the contact site, whereby RhoA is activated and Rac1 is inhibited, driving the paralysis in the membrane and loss of protrusions (Figs. 1c, 2) [5, 30, 69]. In neural crest cells, this switch is dependent upon the activation of the non-canonical Wnt-planar cell polarity (PCP) pathway (Fig. 2c) [5, 69, 70]. Upon a collision many PCP elements, including Dishevelled, Prickle1 and Strabismus, are recruited to the receptor Frizzled7 at the cell–cell contact where their presence is required to drive CIL [5, 10]. The activation of the PCP pathway results in the activation of RhoA, which drives the contraction of the lamellae in a manner dependent on ROCK activity. If ROCK activity is blocked the protrusions fail to collapse at the contact and normal CIL behaviour is lost [5, 10, 69]. In addition, Rac1 activity is inhibited at the contact site, resulting in collapse of the protrusions [29, 70]. This loss of Rac1 activity could in part be due to the antagonistic behaviour that is known to occur between RhoA and Rac1, where the activation of one results in the inhibition of the other [71]. The requirement of RhoA/ROCK activity at the contact site in CIL has also been further established in chick embryonic heart fibroblast where their absence prevents the cells from undergoing CIL, instead they continue migrating in their given direction upon contact as there is no paralysis of membrane ruffles and protrusions [36]. Furthermore, the perturbation of Rac1 in NIH3T3 fibroblasts, either through the use of dominant active Rac1, dominant negative Rac1 or an increase in RhoA activity, results in the loss of CIL when they confront chick heart embryonic fibroblasts [72]. As well as its inhibition downstream of PCP signalling, the inhibition of Rac1 is also driven by the formation of N-cadherin junctions at the contact in the neural crest (Fig. 2b). Blocking N-cadherin, either by antisense morpholino or blocking antibodies, results in a loss of CIL due to an increase in Rac1 activity at the contact driving protrusions at this site [30]. In addition, the overexpression of E-cadherin in the neural crest also results in an increase in Rac1 activity at the contact [33]. Furthermore, these E-cadherin overexpressing cells no longer undergo CIL.

The precise mechanism by which N-cadherin leads to the activation of RhoA and inhibition of Rac1 remains unknown although there are many possibilities (Fig. 2a, b). One possibility is through p120-catenin, which binds to N-cadherin and regulates its turnover [73]. Cytosolic p120-catenin can enhance protrusion formation through the activation of Rac [74, 75]. Interestingly, when it is sequestered to the cell–cell adhesion complex it can no longer promote the activation of Rac and protrusions are inhibited [74]. During CIL N-cadherin could be sequestering p120-catenin preventing it from activating Rac at the contact. Furthermore, the elevation of Rac1 at the contact in neural crest cells overexpressing E-cadherin appears to be dependent on its interaction with p120-catenin and when this interaction is blocked Rac1 activity is once again reduced at the contact [33]. This suggests the ability to prevent p120-catenin from activating Rac1 is specific to the way it is sequestered by N-cadherin. p120-catenin has also been implicated in modulating RhoGTPase activity downstream of Wnt signalling [76–78]. It is also possible that p120-catenin may be modulating the activity of Rho and Rac at the contact after activation of the PCP pathway. The RhoGTPase switch that occurs at the contact upon a collision could also be mediated by the inhibition of the GEF-Trio at this site. Trio can activate Rac1 and modulate the activity of RhoA. It localises to the cell–cell contact in the neural crest in vivo, downstream of the polarity protein Par3, where its inhibition appears to be required for CIL [37]. Furthermore, there is evidence that Trio is recruited downstream of cadherin-11 (Fig. 2a) and its inhibition could provide a mechanism for RhoA activation and Rac1 inhibition upon a collision [79]. It is likely the cadherins recruit Par3 to the contact where it inhibits Trio, resulting in the inhibition of Rac1. An additional mechanism driving the RhoGTPase switch is through the interaction between the nucleotide diphosphate kinase–nm23, and the GEF-Tiam1 that activates Rac1 (Fig. 2b). Nm23 has been identified at the cell–cell contact site in glial cells undergoing CIL where it is localised to N-cadherin [14]. At the cell–cell contact nm23 associates with Tiam1 and inactivates it resulting in the inhibition of Rac1 at this site. EphA/ephrinA signalling leads to RhoA/ROCK activation at the contact (Fig. 2d) [16], via the GEF-Vav2, which is recruited to EphA when it is activated upon binding ephrinA [35]. Furthermore, it has recently been discovered that Rac1 activity in the overlapping protrusions of colliding fibroblasts is regulated by the GAP srGAP2 [80]. It appears that slit-robo signalling is activated in overlapping protrusions during a collision resulting in the activation of srGAP2 and the localised regulation of Rac1 activity [80]. This localised signalling event is required to prevent the cells continued migration and drive the repolarisation of the cells. Each of these different mechanisms regulating small GTPases during CIL could happen in distinct cells or in the same cell. If they occur in the same cell the net balance of all these molecular interactions will determine the final outcome and if a cell undergoes a CIL response.

#### Microtubules upon a collision

In addition to their role in regulating the actin cytoskeleton, RhoGTPases also play an essential role in the regulation of microtubules. Microtubules are stabilised in the leading edge where they are important for maintaining the polarity of a cell and driving directional migration [81, 82]. Stabilised microtubules promote membrane ruffling and the formation of lamellipodia [81], whilst inhibiting contractility through the down regulation of stress fibre and focal adhesion formation [83]. Furthermore, microtubules help maintain cell–cell adhesion complexes [84]. In haemocytes, microtubule bundles are observed in the leading edge where they stabilise the protrusion [6]. When two haemocytes collide the microtubule bundles align across the two colliding cells [6], this coincides with a deceleration of the cells during CIL [48]. It is likely the alignment of microtubule bundles in colliding haemocytes plays a role in the inhibition of the forward movement of the cells, potentially by generating a physical barrier that prevents the cells’ continued migration. If the microtubules cannot be stabilised then polarity is lost in the haemocytes and they no longer undergo CIL [6]. It is possible that the initial coupling of microtubules in colliding cells promotes the formation of the cell–cell adhesion complex that is required to drive CIL.

### The cells repolarise and new protrusions form away from the contact

#### Rac1 activity away from the contact

Another key feature of CIL is the repolarisation of the cells away from the contact after a collision (Fig. 1d). The repolarisation of colliding cells requires a switch in front–rear polarity. In order for this switch to occur not only does RhoA have to be elevated and Rac1 inhibited at the contact, as discussed above, but a new leading edge must form away from the contact. The formation of a new leading edge is dependent on the interplay between adhesions, RhoGTPases and the cytoskeleton. This requires the increase in Rac1 activity away from the contact driving the formation of lamellipodia in this region [33, 85]. During collisions of neural crest cells the switch in the localisation of Rac1 activity has been visualised [33]. In a free migrating cell Rac1 is activated in the leading edge of the cell. Upon a collision Rac1 is inhibited at the contact and subsequently becomes active away from the contact [33]. An elegant experiment in the neural crest recently showed the importance of Rac1 activity in the leading edge after a collision. Cells overexpressing E-cadherin, where Rac1 activity is increased near the contact, do not separate after colliding. However, the activation of photoactivatable Rac1 in the free edge of a cell is sufficient to promote the separation of the cells [33]. This is of particular interest as it suggests the repolarisation of the cells away from the contact is enough to drive separation of the cell even when Rac1 activity is elevated at the contact due to the presence of E-cadherin.

#### Microtubule dynamics

In addition to a switch in Rac1 activity, a switch in the dynamics of microtubules is also required to drive the repolarisation of cells after a collision [36, 37, 86]. Microtubules are stabilised in the leading edge of a cell where they are required to reinforce its polarity [81, 82]. Upon a collision there is a change in the dynamic behaviour of the microtubules at the site of contact, with an increase in the frequency of catastrophe events and rates of shrinkage and growth [37]. This increase in dynamic behaviour at the contact is required for CIL [6, 8, 36, 37]. In the neural crest the dynamic behaviour of microtubules seems to be dependent upon the cell polarity protein—Par3 [37]. Par3 localises to the cell–cell contact where it promotes microtubule catastrophe through the inhibition of the GEF-Trio and subsequent inhibition of Rac1. In haemocytes microtubule bundles align between colliding cells upon a collision and their subsequent collapse is required for a normal CIL response [6]. In addition to an increase in their dynamics at the contact site, microtubules also become stabilised away from the contact further driving the repolarisation of the cell [86].

### The cells separate and migrate away from each other

#### Tension build-up across the contact

**Fig. 3:**
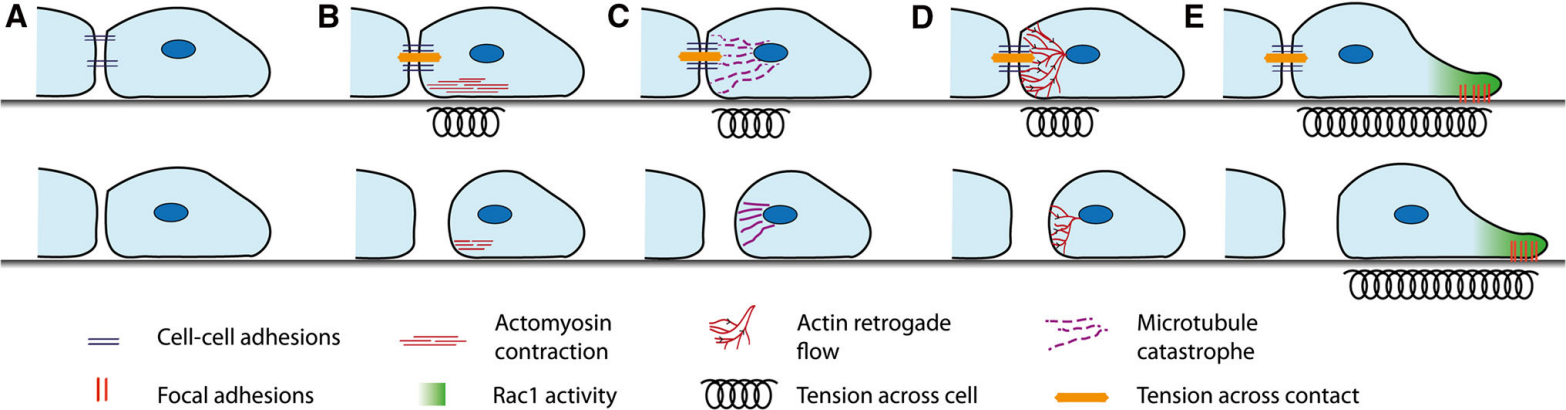
Possible mechanisms stimulating the separation of the colliding cells. a The cell–cell adhesions disassemble or become internalised. This could be triggered by either an addition of tension or a signalling event. The disassembly of the contact between the cells would break the contact and cause the cells to separate. b ROCK activates Myosin II that drives actomyosin contraction near the contact site. This contraction could generate tension across the contact and pull the cells apart. c Microtubules at the contact can restrict the membranes dynamics and give stability to the contact site. If microtubules undergo a sudden catastrophe event this would increase tension across the contact site and this could be sufficient to force the cell–cell adhesions apart causing the cells to separate. d The continuous retrograde flow of actin can generate tension in the lamellae and across both cells when they are coupled through the cell–cell adhesions. This tension could build until it becomes so great it snaps the cell–cell adhesions apart causing the cells to separate. e The repolarisation of the cell away from the contact, driven by Rac1 activity and focal adhesions stabilising the new protrusions, can generate tension across the whole cell. This could be sufficient to drive the separation of the cells

The driving force behind the cells’ separation after a collision is still not fully understood (Fig. 3). It has long been established that there is a build-up in tension across the contacting lamellae [25, 28, 48, 50]; however, how this tension builds up and whether this tension alone is sufficient to tear apart the contacting cells remains unknown. In haemocytes a sudden retraction of lamellae is observed as the cell–cell adhesion complex is broken and the tension across the complex is released [48]. There is much speculation as to what triggers separation and we shall discuss the possibilities.

One possible event that could be triggering the separation of cells after a collision is the disassembly or internalisation of the cell–cell adhesion complex (Fig. 3a). This would uncouple the cells and release the tension across the contact causing the cells to come apart. An alternative possibility is that tension is built up to such a degree across the contact that it forces the cell–cell adhesion apart. This tension could be generated through various means. The activation of RhoA and subsequently ROCK at the contact upon a collision [5, 16, 69] was believed to trigger actomyosin contraction. Actomyosin contraction in the contacting lamellae would result in tension being generated across the contact (Fig. 3b). Myosin II coated stress fibres align between colliding haemocytes and mutants that are lacking in myosin II show a reduction in lamellae tension in the contacting lamellae [48]. It has been hypothesised that myosin-driven contraction of these stress fibres could be sufficient to drive the separation of the cells. Interestingly, however, there is evidence that RhoA/ROCK activation at the contact site does not act through actomyosin contraction as normal CIL behaviour can still occur when myosin contraction is blocked through the use of blebbistatin [36]. It appears instead that RhoA/ROCK activity acts through the mediation of microtubule dynamics [36]. Upon a collision an increase in microtubule dynamics and catastrophe events is required for CIL [6, 8, 36, 37]. Thus, a microtubule catastrophe event could trigger the separation of the cells after a collision by causing a sudden increase in tension across the contact that may be sufficient to force the contact apart (Fig. 3c).

The coupling of the actin cytoskeletons in colliding cells can also generate tension by linking the actin retrograde flow in the lamellae of both cells via cell–cell adhesions across the contact. In a mechanism similar to integrin, the cell–cell adhesions act as a clutch by anchoring the cytoskeleton to a point of resistance [48, 49, 87]. This causes a deceleration of the continuous actin retrograde flow and results in a build-up of tension across the cell–cell contacts and in the lamellae, as actin retrograde flow continues to generate a force that is pulling the cells away from each other. This actin retrograde flow alone could generate enough tension across the cell–cell contacts that a point is eventually reached where the force is too great and the cell–cell adhesion is pulled apart (Fig. 3d).

In addition, the repolarisation of the cells as a whole is necessary for the separation of the cells after a collision (Fig. 3e) [33, 86]. The neural crest cell–cell adhesion complexes remain intact when protrusions are inhibited from forming away from the contact due to physical constraint [33]. This suggests the cells need to pull apart from each other in order for the cell–cell adhesions to be lost. Furthermore, stimulating protrusion formation through the use of a photoactivatable Rac1 in the free edge of cells overexpressing E-cadherin, which do not separate upon a collision, is sufficient to drive the separation of these cells [33]. This indicates that neural crest cells start migrating away from each other prior to the loss of the cell–cell adhesions and this pulling apart is necessary and sufficient to drive the breakdown of these adhesions.

It appears that a variety of mechanisms (Fig. 3) may be stimulating tension generation across the contact and the disassembly of cell–cell adhesions. Each event alone may not be sufficient to drive the separation of the cells, but together they generate enough force and possibly stimulate a signalling event that results in the disassembly of cell–cell adhesions and the subsequent separation of the cells. It is unclear how cell dependent the precise mechanism of separation is, or whether it is conserved across different cell types. A more thorough examination of this event is required to fully understand what drives the separation of cells after a collision.

#### Cell–matrix adhesions

Cell–matrix adhesions play a core role in cell migration and therefore are central to CIL. Cell–matrix adhesions form a transmembrane complex that crosslinks the extracellular matrix to the intracellular cytoskeleton via integrins and adapter proteins. This generates a physical connection linking the external environment to the cytoskeleton and results in force generation and cytoskeletal rearrangements. In addition, this link can also induce internal signalling that can be stimulated by the external environment. The behaviour of cell–matrix adhesions during CIL was first speculated upon by Abercrombie [23], although their behaviour and importance during this process is still not fully understood. Cell–matrix adhesions were first characterised during CIL by Harris where he observed a detachment of cell–matrix adhesions in the lamellae upon a collision. This would lead to the cell–cell contact coming under tension once these adhesions to the substrate were lost [50]. The cells would subsequently separate after the complete loss of cell–matrix adhesions [50]. Interestingly however, Abercrombie noted a conflicting observation using interference reflection microscopy [38], a method that assumes strong cell–matrix adhesions occur where the cell membrane is at its closest to the substrate [88]. Using this imaging technique to infer where cell–matrix adhesions are, Abercrombie concluded that adhesions to the substrate actually persist during a collision even when the lamellae contract [38]. These apparent contradictory results have not being revisited in the 40 years since these observations, and it is still unknown what happens to the cell–matrix adhesions upon collision and if they play a role in driving separation.

Integrin signalling has been identified in myoblasts where ectopic expression of either α5 integrin, β1 integrin or downstream effectors of integrin—such as paxillin and FAK—results in a paralysis of membrane ruffling and lamellae activity upon a collision [56]. There is further evidence of cell–matrix adhesions during CIL in the neural crest. Syndecan-4, a transmembrane heparan sulphate proteoglycan that can crosslink the extracellular matrix to actin via the adapter protein α-actinin [89] and stimulate focal adhesion formation [90], is essential for the directional migration of the neural crest in vivo [69]. In addition, the loss of syndecan-4 results in a loss of CIL with protrusions no longer inhibited towards the contact, as in the case in control cell, due to a huge increase in Rac1 activity across the whole cell periphery. This suggests the presence of syndecan-4 inhibits Rac1 activity at the contact, although where syndecan-4 is localised in the neural crest or how it inhibits Rac1 activity has not yet been identified. In fibroblasts, however, there is evidence that syndecan-4 regulates Rac1 activity through the mediation of PKCα, which plays a role in localising Rac1 activity to the leading edge [91]. Integrin-based cell–matrix adhesions have been visualised in the neural crest [10, 33]. Interestingly, they show a distinct difference in morphology in the free edge versus the site adjacent to the contact. Large elongated adhesions are observed in the free edge, whereas the adhesions near the contact are much smaller and rounded in shape. Interestingly, these small adhesions near the contact become enlarged when E-cadherin is overexpressed [33]. Whether this enlargement is a contributing factor or just a consequence of the loss of CIL in E-cadherin overexpressing cells is unknown.

Cell–matrix adhesions are important mediators of actin retrograde flow rates [92]. The engagement of these adhesions slow actin retrograde flow by generating friction between the actin network and the substrate, consequently generating traction [87]. Changes in actin retrograde flow during CIL have recently been visualised in haemocytes in vivo [48]. It is possible these changes are not solely due to the engagement of the cell–cell adhesion complex, as discussed above, but also driven by changes in cell–matrix adhesion behaviour. It would be of interest for cell–matrix adhesions to be imaged in this in vivo model so their dynamics during CIL can be understood.

## Concluding remarks

CIL is a complex process that requires careful coordination of the cell–cell adhesions, cell–matrix adhesions, activity of the RhoGTPases and cytoskeleton dynamics. Perturbing any of these factors disrupts CIL; the cessation of movement in the direction of contact. Although its importance in vivo is only just beginning to be revealed, CIL has already been identified as the driving force behind the collective directional migration of the neural crest [5, 10], the precise patterning of haemocytes in *Drosophila* [17] and the regular dispersion of Cajal–Retzius neurons throughout the cortex [7]. In addition, CIL can promote the invasion of metastatic cells [15] and its loss towards healthy tissue has long been established as a sign of malignancy although, as yet, it remains unobserved in vivo [11–14, 16, 44]. Although many molecular mechanisms and components of CIL have been identified the precise role and regulation of many others are still not fully understood. One outstanding question is the driving force behind the separation of the cells after a collision. Another is the role of cell matrix adhesions during CIL. Thanks to its discovery in the embryo and advances in imaging techniques, these questions regarding CIL should be answered in the near future.
